# The Blast Fungus Decoded: Genomes in Flux

**DOI:** 10.1128/mBio.00571-18

**Published:** 2018-04-17

**Authors:** Thorsten Langner, Aleksandra Białas, Sophien Kamoun

**Affiliations:** a The Sainsbury Laboratory, Norwich Research Park, Norwich, United Kingdom

**Keywords:** Magnaporthe oryzae, blast fungus, gene flow

## Abstract

Plant disease outbreaks caused by fungi are a chronic threat to global food security. A prime case is blast disease, which is caused by the ascomycete fungus Magnaporthe oryzae (syn. Pyricularia oryzae), which is infamous as the most destructive disease of the staple crop rice. However, despite its Linnaean binomial name, M. oryzae is a multihost pathogen that infects more than 50 species of grasses. A timely study by P. Gladieux and colleagues (mBio 9:e01219-17, 2018, https://doi.org/10.1128/mBio.01219-17) reports the most extensive population genomic analysis of the blast fungus thus far. M. oryzae consists of an assemblage of differentiated lineages that tend to be associated with particular host genera. Nonetheless, there is clear evidence of gene flow between lineages consistent with maintaining M. oryzae as a single species. Here, we discuss these findings with an emphasis on the ecologic and genetic mechanisms underpinning gene flow. This work also bears practical implications for diagnostics, surveillance, and management of blast diseases.

## COMMENTARY

Magnaporthe oryzae (syn. Pyricularia oryzae) is a multihost pathogen that not only infects wild grass species but also cereal crops such as rice, wheat, barley, oat, and millet, and consequently, it destroys food supplies that could feed hundreds of millions of people (1). The propensity of this pathogen to occasionally jump from one grass host to another, combined with global trade and climate change, have resulted in increased incidence of blast diseases. One example is blast disease of wheat, which was first reported in Paraná State, Brazil, in 1985, and has since gone pandemic, threatening a staple crop critical to global food security (2, 3).

**FIG 1:**
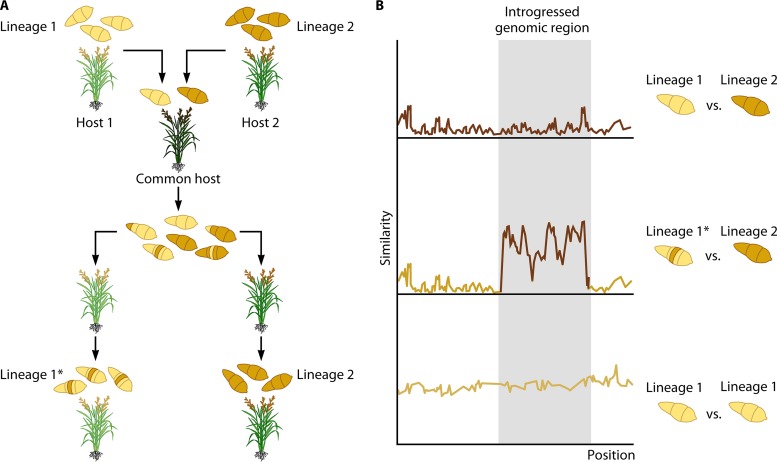
Coinfection on common host plants may facilitate genetic exchange between distinct Magnaporthe oryaze lineages. (A) Although the M. oryzae species complex has the capacity to infect a wide range of grass species, individual lineages tend to be host specific and infect only a limited number of hosts, e.g., lineage 1 (yellow) and lineage 2 (brown) infect host 1 (light green) and host 2 (green), respectively. However, under certain ecologic conditions, two distinct lineages may occasionally colonize or jump on a common host (dark green). Coinfection on a shared host enables interlineage genetic exchange, such as recombination and gene flow, leaving footprints in pathogen genomes. Subsequent selection pressure imposed by the original hosts can either counterselect introgressed segments (lineage 2) or lead to the emergence of a new (sub)lineage (lineage 1*). (B) Similarity plot between different M. oryzae lineages with a genomic region from lineage 2 introgressed into lineage 1, leading to the emergence of lineage 1* (gray box).

It is against this backdrop that Gladieux and colleagues published a much-anticipated study of M. oryzae genetic structure and how this structure relates to its host range (first posted in bioRxiv on 10 July 2017) (4). They report a comprehensive population analysis of M. oryzae based on genome sequences of 76 isolates from 12 grass host genera. Their first contribution is to reject the prior classification of a subset of wheat isolates as a separate species based on analyses of 10 concatenated gene sequences (5). The previously described pattern that led to this taxonomic slip can be explained by the uneven distribution of alleles of 1 of the 10 genes, *MPG1*, across the M. oryzae lineages, which becomes clear when a larger set of isolates is examined. The genome-wide analyses of Gladieux and colleagues (4) are concordant with a single-species model, with M. oryzae consisting of an assemblage of fairly well differentiated lineages that nonetheless retain a measurable degree of recombination and gene flow. A pattern of incipient speciation driven by reproductive isolation on specific hosts is counteracted by the capacity of distinct lineages to colonize or jump to common host plants, thus setting the stage for the occasional genetic exchanges (Fig. 1A).

The discovery that interlineage genetic exchanges are widespread in M. oryzae has important consequences for understanding the evolutionary potential of this pathogen. The work of Gladieux and colleagues expands on previous anecdotal documentation of gene flow in the blast fungus. In 2011, Chuma et al. proposed the concept of “mobile effectors” based on horizontal transfer of the Avr-Pita effector genes between M. oryzae lineages—a process that enables asexual lineages to recover deleted genes (6). More recently, Inoue et al. flagged two somewhat distantly related Brazilian isolates of M. oryzae as having identical sequences of the PWT3 effector (3). Comparative genome analyses revealed that a 1.6-Mbp segment, containing *PWT3*, has probably been transferred from *Brachiaria* isolate Br35 to *Triticum* (wheat) isolate Br48, thus enabling Br48 to infect wheat plants that carry the resistance gene *RWT3* (Fig. 1B). Therefore, gene flow in M. oryzae can be a consequential evolutionary mechanism.

Other important topics raised by the study of Gladieux and colleagues (4) include the ecologic and genetic mechanisms underpinning gene flow. What are the ecologic factors that modulate rates of genetic exchange within M. oryzae? M. oryzae is a pathogen of the Poaceae (grasses), one of the most successful plant families in terms of abundance, species richness, and ecologic dominance (7). Grasses dominate ecosystems that add up to about one-third of the land surface of earth (8). The ubiquity of the grass hosts might create an ecologic framework that promotes coinfection by distinct lineages of M. oryzae, thus countering strict lineage reproductive isolation and slowing down incipient speciation. Unsurprisingly, the rice-infecting lineage is highly differentiated, possibly as a consequence of its domestication along with rice (9). However, the interplay between agricultural and wild ecosystems remains poorly investigated. The paper by Gladieux and colleagues highlights the importance of studying wild pathosystems of the blast fungus in parallel to crop systems to fully understand the evolutionary dynamics of this pathogen. One key question is the degree to which gene flow from pathogens of wild hosts contributes to the emergence of new crop-infecting races as discussed above for wheat blast (3) (Fig. 1A).

What are the genetic mechanisms that underpin gene flow in M. oryzae? Although long considered to be asexual, it is now well accepted that the rice blast fungus undergoes sexual reproduction at least in some parts of its geographic range (10). This is consistent with the emerging view in mycology that fungi are facultative sexual organisms with clonality not as exclusive as previously thought (11). However, some M. oryzae lineages may have lost the capacity to undergo sexual reproduction to become obligate asexuals. This might be the case for the rice-infecting lineage, which is considered to be primarily female sterile and therefore unable to mate with fertile lineages (9, 12, 13). It will be fascinating to identify the genetic basis of M. oryzae loss of fertility, given that the underlying mutations would enhance lineage reproductive isolation and reinforce lineage genetic integrity.

The extent to which nonmeiotic parasexual processes of genetic recombination take place in fungi is a matter of debate (11). However, the possibility that parasexuality contributes to gene flow in the blast fungus needs to be seriously considered. Chuma et al. evoked retroduplication of a retrotransposon and its flanking effector gene as evidence of horizontal transfer between asexual clones (6). At recent international conferences, Peng et al. (14) and Barbara Valent (15) reported discovering accessory minichromosomes from long-read assemblies of the genomes of wheat blast isolates. Remarkably, the minichromosomes differed significantly in size and effector (virulence) gene content between wheat blast strains, indicating that they are more plastic than the core chromosomes in a framework reminiscent of the “two-speed genome” model (16). Future analyses will determine whether these accessory chromosomes facilitate parasexual horizontal gene transfer in M. oryzae similar to what has been described for other plant-pathogenic fungi, such as Fusarium oxysporum (17). In addition, these recent reports highlight the importance of carrying comparative genomics analyses beyond the level of single nucleotide polymorphism (SNP) diversity towards analyzing genomic structural variation, which will most certainly deepen our understanding of genetic diversity of M. oryzae.

The finding that M. oryzae lineages exhibit a degree of genetic mixing bears important implications for applied plant pathology. It is heartening that Gladieux and colleagues (4) do not oversimplify their description of the complex genetic structure of this species. Their high-resolution genetic analysis supports the view that plant pathologists should not be satisfied with diagnostics at the pathogen species level. Genetic diagnostic assays based on one or a few nucleotide polymorphisms are unlikely to provide sufficient resolution to identify M. oryzae lineages and sublineages. There is a need to implement rapid and routine genomic surveillance to monitor new disease outbreaks (18–20). Gladieux and colleagues provide the data set necessary to interpret genomic sequences from emergent epidemic strains.

There are also implications for blast disease management. To which degree do the crop-infecting lineages exchange genetic material with other strains? We need to know the incidence and rate of gene flow in the crop pathogens. As the emergence of wheat blast illustrates, M. oryzae genotypes that infect wild hosts serve as reservoirs that spawn and fuel new disease outbreaks. We need a better understanding of the risk potential of genetic exchanges. For example, what is the likelihood that the Bangladesh wheat blast lineage, which was recently introduced to Asia from South America (2), recombines with endemic strains, and what are the risks associated with such events?

Several additional questions remain unanswered. Gladieux and colleagues (4) stopped short of analyzing variation in gene flow rates between lineages and how that relates to their degree of host specialization. One testable hypothesis is that the more host-specialized lineages, such as the rice-infecting lineage, have experienced reduced frequency of genetic exchanges. It would also be interesting to determine the extent to which natural selection has impacted genetic admixture. What is the identity of the genes that have moved between M. oryzae lineages? Are they enriched in genes that encode adaptive traits, such as effector genes?

The sequencing of the M. oryzae genome in 2005 kicked off the research field of pathogenomics of plant fungi (17, 20). By decoding M. oryzae genomes at the population level, Gladieux and colleagues (4) mark yet another milestone in what has become a very active area of research. These pathogens carry complex and incredibly plastic genomes that enable them to jump from one host to another and crisscross the planet, causing havoc and despair. The study by Gladieux and colleagues demonstrates that we need to understand M. oryzae genomes at the population level to expose a fascinating fungus and tackle a formidable enemy.
